# How to Increase the Nutritional Quality of Stinging Nettle Through Controlled Plant Nutrition^§^

**DOI:** 10.17113/ftb.61.04.23.8119

**Published:** 2023-12

**Authors:** Mia Dujmović, Nevena Opačić, Sanja Radman, Sanja Fabek Uher, Lepomir Čoga, Marko Petek, Sandra Voća, Jana Šic Žlabur

**Affiliations:** 1 University of Zagreb Faculty of Agriculture, Department of Agricultural Technology, Storage and Transport, Svetošimunska cesta 25, 10000 Zagreb, Croatia; 2 University of Zagreb Faculty of Agriculture, Department of Vegetable Crops, Svetošimunska cesta 25, 10000 Zagreb, Croatia; 3 University of Zagreb Faculty of Agriculture, Department of Plant Nutrition, Svetošimunska cesta 25, 10000 Zagreb, Croatia

**Keywords:** sustainable food production, food quality, plant nutrition, bioactive compounds, antioxidant capacity

## Abstract

**Research background:**

As food production faces major challenges, modern agricultural practices are increasingly focused on conserving resources, reducing negative environmental impacts and sustainably producing food with a high content of health-promoting phytochemicals. During production, many factors can affect the quality and chemical composition of a final food product. Proper selection of cultivating conditions, especially a balanced nutrition, can significantly increase nutritional value and result in foods with strong biological and functional properties. Stinging nettle is a rich source of minerals, vitamins, pigments, phenols and other bioactive compounds and can be consumed as a green leafy vegetable with beneficial effects on human health. Therefore, the aim of this study is to determine the nutritional quality and antioxidant capacity of stinging nettle leaves under the influence of different nutrient solution (NS) treatments and three harvest cycles.

**Experimental approach:**

The experiment was conducted in a floating hydroponic system in which treatments with different nutrient solutions were applied and three harvest cycles were carried out. After each harvest, the following treatments were applied: treatment 1 – depletion of nutrient solution by adding water, treatment 2 – supplementation of nutrient solution by adding initial nutrient solution and treatment 3 – correction of nutrient solution by adding nutrients. Among the bioactive compounds, minerals, ascorbic acid, phenols and photosynthetic pigments content, as well as antioxidant capacity were analysed spectrophotometrically, while individual phenols were determined by liquid chromatography.

**Results and conclusions:**

Different nutrition solution treatments and the number of harvest cycles had a significant effect on the content of the analysed bioactive compounds. The highest mass fraction (on fresh mass basis) of total phenols expressed as gallic acid equivalents (377.04 mg/100 g), total flavonoids expressed as catechol equivalents (279.54 mg/100 g), ascorbic acid (112.37 mg/100 g) and pigments (total chlorophylls 1.84, and total carotenoids 0.36 mg/g) as well as the highest antioxidant capacity expressed as Trolox equivalents (35.47 µmol/g) were recorded in the samples supplemented with nutrient solution (treatment NS2) and analysed after the third harvest.

**Novelty and scientific contribution:**

This is the first time that stinging nettle leaves have been produced in a floating hydroponic system by controlled plant nutrition. We have set this type of nutritional manipulation with multiple harvest cycles as an innovative technique for the production of novel food with improved nutritional value that can be consumed as green leafy vegetables.

## INTRODUCTION

Food production faces numerous problems and global challenges due to the pronounced consequences of climate change, which has led to more frequent droughts, floods, natural disasters, soil degradation and arable land loss (*1*). Today, food production is one of the main contributors to global warming through the emission of greenhouse gasses and soil and water pollution through the misuse of chemical fertilizers and pesticides (*2*, *3*). Thus, there is increasing urgency for a stronger focus on adapting the food production chain to sustainable solutions. One of the main objectives of the strategy of European Union and Circular Economy Action Plan (CEAP) (*4*) is to reduce pressure on natural resources and ensure sustainable production. Another challenge for food production is to provide a growing population with an adequate amount of nutrient-rich food.

The nutritional, functional and biological properties of foods of plant origin primarily depend on the content and composition of phytochemicals with significant antioxidant activity. Stinging nettle (*Urtica dioica* L.) is a plant species that has been known for its nutritional and medicinal properties since ancient times (*5*). Nettle has a great potential in food processing for the production of food supplements, tinctures and capsules (*6*) and as a source of green pigments for food colouring (*7*). It is also used in the pharmaceutical, cosmetic and textile industries. All parts of the plant contain large amounts of different bioactive compounds, but leaves are the richest source, which is why they are intended for consumption as a green leafy vegetable (*6*). Nettle leaves contain: carbohydrates, dietary fibre, protein, fatty acids (mostly palmitic and linoleic acids), vitamins (A, B group vitamins, C, D, E and K), minerals (especially P, Ca, Mg and Fe), photosynthetic pigments (chlorophyll a and b, carotenoids), amino acids, organic acids, tannins, terpenoids and phenols (*8*, *9*). Determined individual phenolic compounds are: caffeic, coumaric, chlorogenic, hydroxybenzoic, vanillic and quinic acids, as well as caffeic and quinic acid derivatives and rutin, quercetin and kaempferol (*8*-*10*). The complex chemical composition and antioxidant capacity are responsible for the proven specific biological effects of stinging nettle. Pharmacological studies showed antioxidant, antimicrobial, anti-inflammatory, antidiabetic, hepatoprotective, antitumour (*5*, *8*, *9*, *11*) and other properties of stinging nettle. However, the chemical composition of nettle leaves can vary significantly depending on the origin of the plant material. Nowadays, nettle is usually traditionally collected from the wild, but this plant material is of questionable quality and has inconsistent chemical composition. In addition, nettle can absorb increased amounts of heavy metals and nitrates from the soil, making it unfavourable for consumption (*9*, *12*). Controlling abiotic and biotic factors and managing production conditions can significantly improve the quality and safety of stinging nettle.

The floating hydroponics is a production technique that offers solutions to overcome the above-mentioned problems related to food production. It allows a reduced use of fertilizers and pesticides, which has a strong impact on food safety and prevents food contamination. Continuous control of abiotic factors and more efficient use of water and nutrients can result in higher nutritional quality of the raw material by ensuring favourable chemical composition (*9*, *12*). Floating hydroponics is a soilless technique of growing plants using a nutrient solution that provides precise and balanced plant nutrition according to the specific needs of the plant species. Controlled nutrition can affect the amount of nitrogen, which is important because excessive amounts of nitrogen can pose an environmental problem, but also negatively affect food nutritional quality by reducing the accumulation of phenolic compounds, ascorbic acid and other bioactive compounds (*13*-*15*). A very important advantage of floating hydroponics is also the possibility of multiple harvest cycles, which ultimately leads to higher food production while preserving the nutritional value of the raw material.

Considering the above, the aim of this study is to determine the content of bioactive compounds and antioxidant capacity under the influence of the management of different nutrient solutions and three harvest cycles.

## MATERIALS AND METHODS

### Plant material

Fresh nettle leaves were collected from the cultivated stinging nettle grown at the University of Zagreb, Faculty of Agriculture, Department of Vegetable Crops, Zagreb, Croatia. The experiment was conducted in a floating hydroponic system during the autumn-winter growing season 2021/2022 in a heated greenhouse. The amount of 50 nettle seeds (B&T World Seeds, Paguignan, France) was sown per plate opening in polystyrene boards filled with inert perlite (Europerl d.o.o., Samobor, Croatia), on 26 August 2021. After six days of germination under optimal conditions (temperature 20-25 °C, relative humidity 60-70 %), the boards were relocated into three basins filled with Johnson’s nutrient solution (JNS) containing in mg/L: KNO_3_ 250.99, KH_2_PO_4_ 142.7, K_2_SO_4_ 0, Ca(NO_3_)_2_×4H_2_O 501.5, MgSO_4_×7H_2_O 256.25, 13 % FeEDTA 12.8, H_3_BO_3_ 1.32, CuSO_4_×5H_2_O 0.026, MnSO_4_×4H_2_O 0.79, ZnSO_4_×7H_2_O 0.11 and Na_2_MoO_4_×2H_2_O 0.018; electrical conductivity 1.5 mS/cm, pH=5.8–6.2. JNS was used to prevent excessive accumulation of nitrates because it has a lower nitrate content than other commercial nutrient solutions. Originally, basin 1 and basin 2 were filled with JNS prepared independently of the chemical composition of the water, and basin 3 was filled with JNS adjusted according to the chemical analysis of the water. Since stinging nettle can regrow after harvest, three harvest cycles were carried out manually before flowering: on 3 November 2021, 11 January 2022 and 10 March 2022. After each harvest, treatments with different nutrient solutions (NS) were applied by refilling basins to replace the drop level of JNS. In the first nutrient solution treatment (NS1), water was added to basin 1 (depletion of nutrient solution), in the second nutrient solution treatment (NS2), the initial JNS was added to basin 2 (supplementation of nutrient solution), and in the third nutrient solution treatment (NS3), a chemical analysis was performed, based on which the amount of nutrients was added to basin 3 to match the composition of the initial solution (correction of nutrient solution). Due to the depletion of nutrient solution with water, NS1 contained lower concentration of minerals, especially nitrogen, while the supplementation and correction treatments contained higher concentrations of N and other minerals. Also, greenhouse abiotic factors (air temperature and relative humidity) were monitored regularly throughout the experiment, so plants were grown under controlled conditions. After each harvest mechanical impurities were removed from the plant raw material, leaves were separated from the stems and fresh leaves were immediately analysed.

### Determination of physicochemical properties

According to the CIELAB method, the chromaticity parameters of the fresh nettle leaves (L*, a*, b*, *C* and *h*°) were measured using a colorimeter (ColorTec PCM+; PCE Instruments, Southampton, UK). Fifteen leaves were measured, with five leaves representing one replicate, for a total of three replicates per treatment. The L* value represents the lightness of the sample from black to white on a scale from 0 to 100 where 0–50 indicates dark and 51–100 indicates light, while a* and b* values represent chromaticity, *i.e.* the presence of red or green and blue or yellow, respectively.

Using a standard laboratory procedure according to the AOAC International (*16*), total dry matter content (DM/%) was determined by drying in an oven at 105 °C to constant mass. Potentiometric titration with sodium hydroxide solution (*c*=0.1 mol/L) was used to determine total acid content (TA/%) according to AOAC (*16*). To determine total dry matter and acid content, six measurements were made with two measurements representing a replicate, for a total of three replicates per treatment.

### Determination of mineral composition

The dry matter of plant leaves was determined gravimetrically in a drying oven (ST-360T; INKOLAB d.o.o., Zagreb, Croatia) at 105 °C to constant mass (*17*). The dry samples were digested in a microwave oven (ETHOS 1; Milestone S.r.l., Sorisole, Italy) with concentrated HNO_3_ and HClO_4_ acids. In digested samples calcium, magnesium, iron, zinc, manganese, copper and molybdenum were determined by atomic absorption spectrometer (Solar M5; Thermo Fisher Scientific, Winsford, UK), while boron was determined spectrophotometrically (Evolution 60S; Thermo Fisher Scientific) using azomethine-H method (*16*). All analyses were carried out in triplicates.

### Determination of ascorbic acid content

Titration with 2,6-dichlorophenolindophenol (DCPIP) was used to determine the ascorbic acid (AA) content according to the standard AOAC method (*16*). The plant material (approx. 3.5 g samples) was mixed and homogenised with 100 mL of *φ*(oxalic acid)=2 % and filtered through a Whatman filter paper no. 591. A volume of 10 mL of the filtrate was titrated with freshly prepared DCPIP until a specific pink colour appeared that remained stable for several minutes. Analyses for all treatments were carried out in three replicates. The final AA content of the tested samples (in mg/100 g) was calculated according to the following equation:

*w*(AA)=(*V*·f/*m*)·100 /1/

where *V* is the volume of used DCPIP (mL), f is the factor of the DCPIP (mg/mL) and *m* is the sample mass in the filtrate used for titration (g).

### Determination of phenolic compounds

For determination of the total phenolic compounds, the plant material was first extracted with *φ*(ethanol)=80 %. The extraction was performed according to the following procedure: samples (10.00±0.01) g were homogenised with 40 mL of 80 % EtOH, heated to boiling point and refluxed for 10 min. After filtration, another 50 mL of 80 % EtOH were added and the process was repeated as described by Dujmović *et al.* (*18*). The extracts were used for the determination of total phenolic (TPC), total flavonoid (TFC), total non-flavonoid (TNFC) content and antioxidant capacity.

Polyphenolic compounds were determined according to the method described by Ough and Amerine (*19*), based on the reaction with Folin-Ciocalteu reagent, performed in triplicate. For TPC determination, the reaction mixture was prepared in a 50-mL volumetric flask in the following order: 0.5 mL extracts, 30 mL distilled water, 2.5 mL *V*(freshly prepared Folin-Ciocalteu reagent):*V*(water)=1:2 and 7.5 mL saturated Na_2_CO_3_ solution and made up to the mark with distilled water. The prepared reaction mixture was left for 2 h at ambient temperature with occasional shaking and then the absorbance of the extracts was determined spectrophotometrically (1900i; Shimadzu, Kyoto, Japan) at a wavelength of 750 nm using distilled water as a blank. Gallic acid was used as an external standard, and TPC was expressed in milligrams of gallic acid equivalents (GAE) per 100 g fresh mass.

For the determination of TNFC, chemical reactions were performed as follows: 10 mL ethanolic extract, 5 mL *V*(HCl):*V*(EtOH)=1:4 and 5 mL formaldehyde (p.a.) were placed into a 25-mL volumetric flask and the obtained solutions were blown with nitrogen. Further procedure followed the protocol described by Dujmović *et al.* (*18*). TFC was calculated as the difference between the amount of TPC and TNFC and expressed in milligrams of catechol equivalents (CCE) per 100 g fresh mass.

### Identification and quantification of individual phenols by high-performance liquid chromatography

Following the modified method described by Otles and Yalcin (*20*), high-performance liquid chromatography (HPLC) was used for separation, identification and quantification of individual phenolic compounds from fresh stinging nettle leaves. Among the individual phenolic compounds, caffeic, coumaric, ellagic and ferulic acid and naringin were analysed. Phenols were extracted by homogenisation of (1.00±0.01) g fresh leaves with 10 mL of *φ*(MeOH)=80 % using a laboratory homogeniser (UltraTurax T-18; IKA, Staufen, Germany) and further homogenised in closed vessels in an ultrasonic bath (RK 103H; Bandelin, Berlin, Germany) at 50 °C for 30 min. After the first filtration of the extracts through Whatman filter paper no. 591, they were filtered again through Chromafil polyamide filters. The HPLC analyses of phenolic compounds were performed using LC Nexera (Shimadzu) equipped with a photodiode array and fluorescent detector (RF-20Axs), an automatic injector and LabSolution software. Selected individual phenolic compounds and their standards were separated on a Nucleosil® 100-5 C18 (5 µm, 250 mm×4.6 mm i.d.) column (Macherey-Nagel, GmbH, Düren, Germany). Analytical conditions, with minor modifications, were set up according to Repajić *et al.* (*21*) using the same mobile phases, flow rate and applied sample volume. The analyses were performed at 23 °C and the duration of the single run was 45 min. The gradient profile (A/B)/% was as follows: at 0 min 90/10, at 25 min 60/40, at 30 min 30/70 and then after 35 min to 45 min 90/10. All determinations were performed in duplicate. Phenols were detected at wavelengths ranging from 220 to 360 nm and identified based on their retention times compared to commercial standards (Sigma-Aldrich, Merck, Steinheim, Germany). Quantification of individual phenolic compounds was carried out by calculating the external standard using the equation based on the calibration curves for caffeic acid, coumaric acid, ellagic acid, ferulic acid and naringin, respectively:

y=13159.9∙x+12112.7 R^2^=0.9998 /2/

y=2551.11·x+2349.01 R^2^=0.9999 /3/

y=33829.1·x–6862.97 R^2^=1.0000 /4/

y=27461.5·x–90059.3 R^2^=0.9870 /5/

y=3941.28·x–30329.7) R^2^=0.9914 /6/

and was expressed as mass fraction (mg/100 g). The calibration curves were generated using different concentrations (2, 10, 40 and 100 µg/mL) of the standard solution mixture, also injected in duplicate.

### Determination of the photosynthetic pigment content

For the determination of photosynthetic pigments, approx. 0.2 g of fresh leaves were weighed and mixed with a total volume of 15 mL of acetone (p.a.) in three steps using a laboratory homogeniser (UltraTurax T-18; IKA) as previously described by Dujmović *et al.* (*18*). Pigment compounds were determined according to the method described by Holm (*22*) and Wettstein (*23*). Total chlorophylls (TCh), chlorophyll a (Chl_a), chlorophyll b (Chl_b) and total carotenoids (TCa) were determined in three replicates and absorbance values were measured spectrophotometrically (1900i; Shimadzu) at wavelengths of 662, 644 and 440 nm using acetone (p.a.) as a blank. The pigment content was calculated by including the measured absorbance values in the Holm–Wettstein equations below and the results were expressed in mg/g:

*w*(Chl_a)=9.784·*A*_662 nm_−0.990·*A*_644 nm_ /7/

*w*(Chl_b)=21.426·*A*_644 nm_−4.65·*A*_662 nm_ /8/

*w*(TCh)=5.134·*A*_662 nm_+20.436·*A*_644 nm_ /9/

*w*(TCa)=4.695·*A*_440 nm_−0.268·TCh /10/

### Determination of antioxidant capacity

Antioxidant capacity was determined by two different methods: the ABTS assay previously described by Miller *et al.* (*24*) and the FRAP assay according to Benzie and Strain (*25*). The same ethanolic extracts prepared for the detection of phenols were also used for the determination of antioxidant capacity. Measurements for both methods were performed in three repetitions. All chemical materials: ABTS (2,2’-azinobis(3-ethylbenzothiazoline-6-sulfonic acid)), TPTZ (2,4,6-tripyridyl-*s*-triazine), and Trolox (6-hydroxy-2,5,7,8-tetramethylchroman-2-carboxylic acid) were purchased from Sigma-Aldrich, Merck (St. Louis, MO, USA).

The ABTS assay is a colorimetric method that measures the ability of antioxidants to scavenge the generated ABTS^•+^ radical cation compared with a Trolox (water-soluble vitamin E analogue) as an equivalent antioxidant standard. Trolox stock standard solution (2.5 mM) was prepared in *φ*(ethanol)=80 %. The ABTS^•+^ radical cation was generated a day before the analyses by mixing 88 µL of a 140 mM K_2_S_2_O_8_ and 5 mL of a 7 mM ABTS solution and allowed to stand at room temperature in the dark for 16 h. On the day of the analyses, the ABTS solution was prepared as described by Dujmović *et al.* (*18*). The addition of the antioxidants (sample extracts) reduced the ABTS^•+^ to ABTS and the reaction was manifested by discolouration of the blue–green solution. After 5 min of incubation at ambient temperature, the absorbances were measured spectrophotometrically (1900i; Shimadzu) at 734 nm. As a blank, 96 % ethanol was used and final results were calculated based on a calibration curve and expressed as µmol of Trolox equivalents (TE) per gram of sample.

The Fe(III) reducing antioxidant power (FRAP) assay measures antioxidant potential in samples through the reduction of Fe(III) to Fe(II) ion at pH=3.6, reducing colourless Fe(III)-tripyridyltriazine (Fe(III)-TPTZ) to an intensely blue coloured Fe(II)-tripyridyltriazine complex (Fe(II)-TPTZ). Fresh working FRAP reagent was prepared by mixing 0.3 M acetate buffer, a solution of 10 mM TPTZ reagent in 40 mM HCl, and 20 mM FeCl_3_·6H_2_O in a ratio 10:1:1. For reaction, distilled water (960 µL), sample extracts (320 µL) and FRAP reagent (8320 µL) were well mixed. The same reactions were prepared for the blank, except that 80 % EtOH was used instead of the sample extract. The prepared mixtures were incubated for 5 min at 37 °C in a water bath, while absorbance was measured spectrophotometrically (1900i; Shimadzu) at 593 nm. Trolox was used as the antioxidant standard and stock standard solution (2 mM) was prepared in 80 % ethanol. Results were expressed as µmol TE per g of sample.

### Statistical analyses

The cultivation experiment was set according to the randomized complete block design with three replicates and all laboratory analyses were also performed in triplicate. The obtained results were statistically analysed using PROC GLM in SAS software system, v. 9.3. (*26*), subjected to two-way analysis of variance (ANOVA) and expressed as mean values. Mean values were compared by *t*-test (LSD) and considered significantly different at p≤0.05. Different letters indicate significant statistical differences between samples.

## RESULTS AND DISCUSSION

### Physicochemical properties of fresh stinging nettle leaves

The analysed chromaticity parameters showed the highest L* values measured for nettle leaves during depletion of nutrient solution by adding water (NS1) after the second and third harvests with an average value of 45.85 (Table 1). These leaves were significantly lighter than all other samples. Negative a* values represent green colour and in all three treatments the greenest leaves were measured after the first harvest and in the depletion treatment (NS1) after the third harvest with an average value of 15.06. Positive b* values were measured in all treatments, indicating the presence of yellow colour. The results of this study showed that among the tested factors, only the treatment with nutrient solution (NS) had a significant effect on L* values, while both individual factors, NS treatment and number of harvests (HN) had a significant effect on a* and b* values. The determination of chromaticity parameters is important because colour is one of the first properties of food products that consumers notice to determine sensory characteristics, flavour, ripeness and freshness (*27*). It is also an initial indicator of photosynthetic pigment accumulation.

**Table 1:** Chromaticity parameters of fresh stinging nettle leaves cultivated under the different nutrient solution treatments and determined after three harvest cycles

Treatment	L*	a*	b*	C*	*h*°
First harvest					
NS1	(44.1±2.3)^ab^	(-15.3±0.7)^c^	(25.3±1.9)^a^	(29.6±2.0)^a^	(121.3±1.1)^cd^
NS2	(43.2±2.4)^abc^	(-14.7±0.6)^c^	(22.6±1.6)^a^	(27.0±1.8)^a^	(123.0±1.3)^bc^
NS3	(43.3±1.5)^abc^	(-15.5±0.9)^c^	(23.6±2.0)^a^	(28.3±2.1)^a^	(122.7±1.65^bc^
Second harvest					
NS1 NS2 NS3	(45.9±4.6)^a^ (40.3±2.0)^bc^ (40.9±2.0)^bc^	(-13.5±1.5)^bc^ (-11.6±2.6)^ab^ (-11.9±2.0)^ab^	(25.3±4.4)^a^ (16.1±3.1)^b^ (17.6±3.9)^b^	(28.7±4.2)^a^ (19.8±4.0)^b^ (21.2±4.4)^b^	(118.4±4.2)^e^ (125.6±0.9)^a^ (124.1±1.3)^ab^
Third harvest					
NS1 NS2 NS3	(45.8±1.8)^a^ (39.9±1.6)^c^ (40.5±1.0)^bc^	(-14.66±0.04)^c^ (-11.7±1.3)^ab^ (-11.1±1.1)^a^	(25.2±1.0)^a^ (17.51±2.1)^b^ (15.7±1.8)^b^	(29.2±0.8)^a^ (21.0±2.4)^b^ (19.2±2.1)^b^	(120.2±1.0)^de^ (123.7±0.3)^abc^ (125.2±0.5)^ab^
ANOVA	0.0447	0.0059	0.0007	0.0011	0.0003
LSD	4.0681	2.4125	4.6124	5.0578	2.499
NS HN	0.0031 ns	0.0242 0.0006	≤0.0001 0.0043	0.0002 0.0023	≤0.0001 -
NS×HN	ns	ns	ns	ns	0.0206

Results are expressed as mean value±standard deviation. Different letters indicate significant differences between mean values. L*=lightness, a*=green–red components, b*=blue–yellow components, C*=chroma, *h*°=hue, NS1=first nutrient solution treatment (depletion), NS2=second nutrient solution treatment (supplementation), NS3=third nutrient solution treatment (correction); significance of factors and their interactions: NS=nutrient solution, HN=number of harvest cycles, NS×HN=nutrient solution and number of harvest cycles; ns=not significant

Dry matter (DM) is accumulated through photosynthesis, by which plants produce sugars, which are then converted to a diverse organic compound that constitutes about 95 % of plant dry mass (*28*). In this study, the highest DM content was observed after the second harvest cycle in NS2 (supplementation, 23.41 %) and NS3 (correction, 23.96 %) treatment (Table 2). The lowest values were measured in all NS treatments after the first harvest, suggesting that DM content can be increased by applying multiple harvest cycles, which is confirmed by Westwood and Mulcock (*29*). Analysis of the significance of the factors showed that both individual factors, nutrient solution (NS) and harvest number (HN), and their interaction (NS×HN) had a significant effect on DM content. The DM content of plant tissues can be affected by several other factors, such as genetic characteristics, ecological factors (temperature and humidity), precipitation, pollution (*30*) and mostly growing conditions, especially biogenic mineral nutrition. Paulauskienė *et al.* (*31*) determined that the leaves of the wild stinging nettle contained 20.48–24.41 % DM depending on the harvest time, while Radman *et al.* (*32*) reported DM amount of 20.11 % in cultivated nettle leaves. Both mentioned DM values are similar to those obtained in this research after the second and third harvest while the DM content after the first harvest was lower regardless of the NS content. This trend might be a consequence of lower accumulation of nutrients such as N and P, which is in accordance with the study of Akter *et al.* (*33*). In terms of green leafy vegetables, stinging nettle can be considered to have high DM content compared to other species such as spinach (6.3–11.2 %) (*34*), leaf turnip (13.4–14.1 %) and kale (14.7–20.6 %) (*29*). Since DM content is an indicator of nutritional potential and food quality, the high DM value obtained in our research shows high nutrient content of nettle leaves. It is worth mentioning that plant materials with a higher DM, *i.e*. lower water content, have a longer shelf life, better storability and sensory characteristics.

**Table 2:** Total dry matter (DM) and total acid (TA) content of stinging nettle cultivated under different nutrient solution treatments and determined after three harvest cycles

Treatment	DM/%	TA/%
First harvest	
NS1	(13.5±0.3)^f^	(0.05±0.01)^ef^
NS2	(15.5±0.3)^e^	(0.06±0.01)^de^
NS3	(15.1±0.3)^e^	(0.08±0.01)^bc^
Second harvest		
NS1	(21.7±0.2)^c^	(0.09±0.01)^bc^
NS2	(23.4±0.7)^a^	(0.07±0.01)^cd^
NS3	(24.0±0.3)^a^	(0.11±0.01)^a^
Third harvest		
NS1	(21.6±0.2)^c^	(0.09±0.01)^ab^
NS2	(22.5±0.4)^b^	(0.04±0.01)^f^
NS3	(17.8±0.2)^d^	(0.07±0.02)^cd^
ANOVA	≤0.0001	≤0.0001
LSD	0.5782	0.0173
NS	≤0.0001	≤0.0001
HN	≤0.0001	≤0.0001
NS×HN	0.0003	0.0003

Results are expressed as mean value±standard deviation. Different letters indicate significant differences between mean values. NS1=first nutrient solution treatment (depletion), NS2=second nutrient solution treatment (supplementation), NS3=third nutrient solution treatment (correction); significance of factors and their interactions: NS=nutrient solution, HN=number of harvest cycles, NS×HN=nutrient solution×number of harvest cycles

Organic acids from plants are a class of nutrients with acidic properties that have numerous beneficial effects on human health, such as anti-inflammatory and immunoprotective effects as well as stimulation of calcium absorption and prevention of neurodegenerative diseases (*35*). The results of this study showed that regardless of the NS treatment, the highest total acid (TA) content was observed after the second, and the lowest after the first harvest (Table 2). The NS treatment had a significant effect on the TA content with the highest values on fresh mass basis observed in NS3 (correction) after the second harvest (0.11 %). Different results can be explained by the complex and different affinity of the minerals from nutrient solutions to the acidic environment, whereas organic acids can make P and Fe complexes more soluble and available to plants, but also reduce nitrate uptake (*36*). The composition and level of organic acids varies in different food and plant tissues depending on the species, cultivar, age of the plant, tissue type, environment pH and stress conditions (*35*). This study showed that organic acid content can also be affected by different NS treatments and number of harvest cycles. The analysis of the significance of different factors and their interactions showed that both individual factors (NS and HN) and the combination of these two factors (NS×HN) had a significant effect on the TA values.

### Mineral composition of fresh stinging nettle leaves

Minerals are important components of the diet, responsible for maintaining health, normal growth and development (*37*), and their specific amounts are required for the proper functioning of body systems. Since they must be consumed through the diet, it is necessary to pay attention to the mineral-rich sources as their deficiency can have noticeable adverse effects on human health (*38*). Leafy vegetables are generally considered a rich source of minerals (*37*), but their amount and bioavailability depend strongly on the vegetable species. Table 3 shows the mass fraction of selected biogenic elements (minerals) in fresh nettle leaves expressed on fresh mass basis. Ca and Mg are important minerals involved in muscle contraction, formation of bones and teeth, decreasing blood pressure and LDL cholesterol and preventing osteoporosis. They are also cofactors in many enzymatic reactions necessary for protein synthesis, nerve function and many others (*39*). The highest Ca requirement is during the growth phase in childhood and during lactation. Regardless of the NS treatment, the highest mass fractions (on fresh mass basis) of Ca (2106 mg/100 g) and Mg (333 mg/100 g) in nettle leaves were found after the second, while the lowest mass fractions were found after the first harvest. NS treatment had a significant effect on Ca and Mg content in fresh stinging nettle leaves, with the highest values measured during NS1 (depletion). Ca and Mg tend to accumulate in more mature than in young plants (*40*), while studies also show that Ca and Mg mass fractions in plant tissues strongly depends on water availability and salinity. Under the conditions of lower water availability and higher salinity, Ca uptake is lower due to reduced absorption and transpiration (*41*). It is worth noting that according to FAO and WHO (*42*), the recommended Ca intake for adult men and women is 1000 mg/day, which can be provided by consuming 47.5–130 g of the nettle leaves obtained in this study. The recommended daily Mg intake is 190–220 for adult women and 224–260 mg/day for adult men (*42*), which can be achieved by consuming 100 g of nettle leaves obtained from all treatments of second and third harvest cycles. Some sources rich in Ca from leafy vegetables are kale and watercress with average Ca mass fractions of 100 and 150 mg/100 g, respectively (*39*), and good Mg sources are spinach and kale with average values of 53 and 30 mg/100 g, respectively (*43*), which is significantly lower than the results for Ca and Mg in fresh stinging nettle leaves obtained in this study. Thus, nettle leaves can also be considered a rich source of Ca and Mg.

**Table 3:** Mineral content of fresh stinging nettle leaves cultivated under different nutrient solution treatments and determined after three harvest cycles

Treatment				*w*/(mg/100 g)				
Ca	Mg	Fe	Zn	Mn	Cu	B	Mo
First harvest									
NS1	(767±16)^d^	(114.7±5.5)^f^	(1.33±0.01)^e^	(0.41±0.01)^d^	(0.13±0.01)^h^	(0.19±0.01)^c^	(0.66±0.05)^d^	(0.03±0.01)^e^
NS2	(863±63)^d^	(129.9±0.2)^ef^	(1.65±0.08)^d^	(0.29±0.01)^f^	(0.50±0.02)^f^	(0.17±0.01)^c^	(0.71±0.02)^d^	(0.03±0.01)^e^
NS3	(813±9)^d^	(136.8±0.2)^e^	(1.97±0.03)^b^	(0.37±0.01)^e^	(1.08±0.02)^b^	(0.21±0.02)^b^	(0.85±0.01)^c^	(0.03±0.01)^de^
Second harvest									
NS1	(2106±147)^a^	(333±7) ^a^	(1.79±0.02)^c^	(0.60±0.01)^b^	(0.16±0.01)^g^	(0.21±0.01)^b^	(0.79±0.06)^c^	(0.05±0.01)^ab^
NS2	(1725±174)^b^	(279±13)^b^	(2.19±0.01)^a^	(0.45±0.01)^c^	(0.85±0.01)^c^	(0.21±0.01)^b^	(1.15±0.03)^a^	(0.04±0.01)^bc^
NS3	(1671±49)^b^	(291±3)^b^	(2.20±0.07)^a^	(0.45±0.01)^c^	(1.27±0.01)^a^	(0.23±0.01)^a^	(1.09± 0.03)^ab^	(0.05±0.01)^a^
Third harvest									
NS1	(1328±25)^c^	(247±9)^c^	(1.00±0.03)^f^	(0.91±0.03)^a^	(0.80±0.01)^d^	(0.13±0.01)^d^	(0.70±0.01)^d^	(0.04±0.01)^d^
NS2	(1237±11)^c^	(220±6)^d^	(2.05±0.03)^b^	(0.39±0.02)^de^	(0.60±0.02)^e^	(0.07±0.01)^f^	(1.03±0.01)^b^	(0.04±0.01)^cd^
NS3	(813±9)^d^	(229±8)^d^	(2.24±0.02)^a^	(0.37±0.01)^e^	(0.80±0.02)^d^	(0.10±0.01)^e^	(0.84±0.01)^c^	(0.03±0.01)^de^
ANOVA	≤0.0001	≤0.0001	≤0.0001	≤0.0001	≤0.0001	≤0.0001	≤0.0001	≤0.0001
LSD	194.4	15.369	0.1017	0.0323	0.0297	0.0148	0.0748	0.0077
NS	≤0.0001	≤0.0001	≤0.0001	≤0.0001	≤0.0001	≤0.0001	≤0.0001	ns
HN	≤0.0001	≤0.0001	≤0.0001	≤0.0001	≤0.0001	≤0.0001	≤0.0001	≤0.0001
NS×HN	≤0.0001	≤0.0001	≤0.0001	≤0.0001	≤0.0001	≤0.0001	≤0.0001	0.0145

Results are expressed as mean value±standard deviation. Different letters indicate significant differences between mean values. NS1=first nutrient solution treatment (depletion), NS2=second nutrient solution treatment (supplementation), NS3=third nutrient solution treatment (correction); significance of factors and their interactions: NS=nutrient solution, HN=number of harvest cycles, NS×HN=nutrient solution×number of harvest cycles; ns=not significant

The iron mass fraction in fresh nettle leaves was significantly affected by the number of harvests and the NS treatments (Table 3), with the highest Fe mass fraction on fresh mass basis, without significant difference, found after the second and third harvest cycles in NS2 (supplementation) and NS3 (correction) with an average value of 2.21 mg/100 g. Fresh spinach and kale, as known sources of high content of Fe, contain on average 2.8 and 1.7 mg/100 g Fe, respectively (*43*), which are similar values to those of the nettle leaves from this study. However, it should be noted that Fe from vegetables has low bioavailability. Although some dietary factors, such as ascorbic acid and proteins, can increase Fe bioavailability, others, such as Ca, phytates and polyphenols inhibit Fe absorption (*44*). Regardless of its bioavailability, Fe is an essential element for almost all living organisms as it participates in a variety of metabolic processes, including oxygen transport, DNA synthesis and electron transport (*38*). Another mineral essential for human metabolism is Zn, whose mass fraction in nettle leaves also significantly varied depending on the number of harvest cycles and NS treatments. Zn mass fraction in nettle leaves was significantly higher after the second and third harvests than after the first harvest regardless of NS treatment. Given the NS treatment, Zn mass fraction on fresh mass basis was the highest after all harvest cycles in NS1 (0.41, 0.60 and 0.91 mg/100 g), where only the water was replenished. As other studies have shown, the Fe and Zn content in plants strongly depends on several factors, such as water availability, pH, salinity, temperature, maturity stage, *etc.* (*40*), with the content of both increasing mainly with higher water availability and lower salinity. For comparison, the nettle leaves produced in this study are a much better source of Zn than some other leafy vegetables such as kale (0.44 mg/100 g) and spinach (0.7 mg/100 g) (*43*). In addition to Fe and Zn, there are other trace elements that the human organism needs in smaller quantities, but which play an important role: manganese, copper, boron and molybdenum. According to the results, Mn (1.27 mg/100 g) and Cu (0.23 mg/100 g) had the highest values after the second harvest during NS3 (correction). The highest mass fractions of B and Mo were also found after the second harvest. The highest mass fraction on fresh mass basis for B was 1.15 mg/100 g in NS2 (supplementation) and for Mo 0.05 mg/100 g in NS3 (correction). Some other researchers studied the mineral content of stinging nettle and also pointed out the high value of nettle leaves as a rich source of minerals (*9*, *32*). This study showed that the tested factors (NS and HN) and their combination (NS×HN) significantly affected the content of each analysed mineral except for Mo, on whose content NS had no effect. The uptake of mineral nutrients by plants can also be influenced by the type of cultivation, temperature, light, oxygen concentration, nutrient concentration and plant development among others. It is important to emphasise that, in addition to these factors, antagonisms between individual macro- and micronutrients and genetic characteristics also play an important role (*37*, *40*, *41*).

### Ascorbic acid content of fresh stinging nettle leaves

Ascorbic acid (AA) is an important free radical scavenger that can prevent their harmful effects on organisms and is a cofactor for numerous enzymes in plant and human metabolism. The strong antioxidant activity of AA is the best-known property, as it is often used to prevent different diseases and as an additive in food storage (*45*). The accumulation of AA in plants can be influenced by various factors such as climatic conditions (especially light intensity and temperature), environmental stress, genotypic differences, cultivation practices, maturity, harvesting methods (*45*) and plant nutrition.

The results of this study showed that regardless of the NS treatment, the highest AA mass fraction was observed after the third harvest cycle and the lowest after the second (Table 4). Considering the NS treatments, the highest AA mass fraction on fresh mass basis was observed after the third harvest cycle in NS2 (supplementation) at 112.4 mg/100 g. This value was about 139 % higher than the lowest values observed after the second harvest cycle in NS2 (47.0 mg/100 g) and NS3 (48.1 mg/100 g). These results can be explained by the effect of N on AA content. As several studies suggest (*13*, *15*), a higher amount of N leads to a decrease in AA. The highest AA content in NS2 (supplementation) after the third harvest could be due to other minerals present in the nutrient solution, since AA is positively related to the concentration of Mg and P (*46*). Radman *et al.* (*15*) also obtained multiple harvests of field-grown nettles and the results confirmed that an increased number of harvests led to an increase in AA content, just as in the present study. Their results showed AA mass fractions ranging from 16.0 to 112.8 mg/100 g, with the highest AA content corresponding to the highest value obtained in our study. The analysis of the significance and interaction of the tested factors showed that the NS treatment had no significant effect on the AA mass fraction, but the number of harvest cycles (HN) and the combination of both factors (NS×HN) showed a very high statistical significance. Based on the results of this study, it is evident that the stress caused by multiple harvest cycles had a greater influence than plant nutrition. The AA mass fraction can also vary a lot depending on the origin of the raw material. In the harvested wild nettles, the AA mass fraction varied from 0.58 to 25.7 mg/100 g (*31*), while Shonte *et al.* (*47*) reported values of 94.7 mg/100 g of AA for field-grown nettles, which are significantly lower values than in our treatments. According to the Dietary Reference Intakes (*48*) developed by the Food and Nutrition Board (Institute of Medicine), the recommended daily intake (RDI) of vitamin C is 75–90 mg/day for adult women and men, which can be achieved by consuming 67–80 g of nettle leaves collected after the third harvest, supplemented with initial NS as determined in this study.

**Table 4:** The mass fraction of ascorbic acid (AA) and phenolic compounds of fresh stinging nettle leaves cultivated under different nutrient solution treatments and determined after three harvest cycles

Treatment	AA/ (mg/100 g)	TPC (as GAE)/ (mg/100 g)		TNFC (as GAE)/ (mg/100 g)	TFC (as CCE)/ (mg/100 g)
First harvest					
NS1	(72.9±2.0)^d^	(163.9±0.8)^f^		(88.1±1.9)^g^	(75.9±2.3)^f^
NS2	(67.1±1.0)^d^	(194.8±2.8)^e^		(99.1±0.7)^ef^	(95.7±2.1)^de^
NS3	(84.1±2.5)^c^	(202.0±0.9)^d^		(104.6±0.8)^d^	(97.4±0.6)^d^
Second harvest					
NS1	(56.1±4.9)^e^	(204.5±1.5)^d^		(115.8±0.2)^c^	(88.7±1.5)^e^
NS2	(47.0±7.8)^f^	(262.1±1.2)^c^		(128.1±0.8)^b^	(134.0±2.0)^c^
NS3	(48.1±1.2)^f^	(267.0±2.0)^c^		(139.4±0.7)^a^	(127.6±2.5)^c^
Third harvest					
NS1	(95.4±3.4)^b^	(300.1±1.8)^b^		(100.6±1.8)^e^	(199.5±3.6)^b^
NS2	(112.4±4.8)^a^	(377±11)^a^		(97.5±0.7)^f^	(280±11)^a^
NS3	(94.6±8.1)^b^	(163.6±0.8)^f^		(99.0±1.2)^ef^	(64.7±1.8)^g^
ANOVA	≤0.0001	≤0.0001		≤0.0001	≤0.0001
LSD	7.5147	6.9634		1.759	7.6369
NS	ns		≤0.0001	≤0.0001	≤0.0001
HN	≤0.0001		≤0.0001	≤0.0001	≤0.0001
NS×HN	≤0.0001		≤0.0001	≤0.0001	≤0.0001

Results are expressed as mean value±standard deviation. Different letters indicate significant differences between mean values. TPC=total phenolic content, TNFC=total non-flavonoid content, TFC=total flavonoid content, GAE=gallic acid equivalents, CCE=catechol equivalents, NS1=first nutrient solution treatment (depletion), NS2=second nutrient solution treatment (supplementation), NS3=third nutrient solution treatment (correction); significance of factors and their interactions: NS=nutrient solution, HN=number of harvest cycles, NS×HN=nutrient solution×number of harvest cycles; ns=not significant

### Phenolic compounds of fresh stinging nettle leaves

Phenolic compounds play an important role in the protection and reproduction of plants, but when ingested through food, they also have many properties beneficial to human health. Their effects are manifested in antioxidant, anti-inflammatory, antimicrobial, anticancer, cardioprotective and antithrombotic properties, as well as protection against chronic and neurodegenerative diseases (*49*). Therefore, it is essential to determine the content and find measures that help increase the amount of phenols in plant-based foods. The phenolic composition of plants is influenced by many factors, such as climate, soil, variety, phenophase, harvest time, cultivation treatment and applied processes (*50*).

According to the results, both tested factors (NS and HN) and their interaction (NS×HN) had a significant effect on total phenolic (TPC), flavonoid (TFC) and non-flavonoid contents (TNFC) (Table 4). Regardless of the NS treatment, the highest TPC and TFC mass fraction was observed after the third harvest cycle with average values, expressed as gallic acid equivalents (GAE), of 280.3 for TPC and 181.3 mg/100 g for TFC. Considering that bioactive substances are increasingly synthesised and accumulated in stressful conditions as a defence mechanism of the plant, it is likely that the amounts of TPC and TFC were induced by the stress due to multiple harvest cycles. Values for TPC, as GAE, ranged from 163.6to 377 mg/100 g, with the highest TPC (377 mg/100 g) and TFC (280 mg/100 g) observed in the plants subjected to the NS2 treatment (supplementation) and analysed after the third harvest cycle. For TPC, this was 130 % higher than in the lowest observed treatments: first harvest NS1 and third harvest NS3. Due to the addition of initial NS and the specific amount of nutrients, supplementation (NS2) and correction (NS3) treatments contained higher contents of N and other minerals. Higher N amounts can lead to a reduction in phenolic content (*14*), which was the case for TPC, TNFC and TFC in the first two harvest cycles. Radman *et al.* (*15*) investigated the phenolic content of conventionally grown nettles in the open-field and found that it ranged on fresh mass basis from 349 to 963 mg/100 g for TPC, which is in agreement with our results. In different studies, the TPC of harvested wild nettle leaves varied widely (*8*, *11*, *31*), confirming that environmental factors and stress conditions strongly influence phenolic composition.

In addition to the total phenolic compounds, some of the selected individual phenols were identified and quantified: caffeic, coumaric and ferulic acids (group of hydroxycinnamic acids), ellagic acid (group of ellagitannins) and naringin (group of flavanone-7-O-glycosides) (Table 5). According to the results, caffeic acid was not determined in the stinging nettle leaves in this research. The mass fraction of coumaric acid was low, in the range of 0.001–0.7 mg/100 g, with the highest amount detected after the first harvest in NS1. Coumaric acid was not determined in the samples from the second harvest. The highest mass fraction of ellagic acid (2.2 mg/100 g) was found after the first harvest in NS3 (correction), which was about 8-fold higher than in NS1 and NS3 after the second harvest. Ferulic acid mass fraction was not significantly different after first harvest in NS1 and NS2 and after the third harvest in NS2, with an average mass fraction of 5.5 mg/100 g. The highest naringin mass fraction was observed after the second harvest in NS1 (8.4 mg/100 g). Of the individual phenolic compounds determined, naringin was the dominant compound, probably because it plays an important role in leaf development (*51*). The statistical significance of the tested factors showed that different NS manipulations had a significant influence on the contents of ellagic and ferulic acids and naringin. The number of harvest cycles had a great impact on the contents of coumaric, ellagic and ferulic acids, and the combination of factors (NS×HN) significantly affected the contents of ellagic and ferulic acids and naringin.

**Table 5:** The mass fractions of individual phenolic compounds in fresh stinging nettle leaves cultivated under different nutrient solution treatments and determined after three harvest cycles

Treatment			*w*/(mg/100 g)		
Caffeic acid	Coumaric acid	Ellagic acid	Ferulic acid	Naringin
First harvest						
NS1	nd	(0.7±0.1)^a^	(1.05±0.05)^b^	(5.6±0.3)^a^	(7.96±0.05)^bc^
NS2	nd	(0.2±0.2)^abc^	(0.8±0.1)^bcd^	(5.39±0.06)^a^	(7.87±0.04)^c^
NS3	nd	(0.6±0.6)^ab^	(2.2±0.2)^a^	(5.1±0.2)^ab^	(8.25±0.05)^ab^
Second harvest						
NS1 NS2 NS3	nd nd nd	nd nd nd	(0.26±0.01)^d^ (0.29±0.01)^cd^ (0.28±0.01)^d^	(3.5±0.1)^c^ (3.46±0.02)^c^ (3.43±0.05)^c^	(8.37±0.03)^a^ (7.96±0.02)^bc^ (7.98±0.03)^bc^
Third harvest						
NS1 NS2 NS3	nd nd nd	(0.1±0.1)^bc^ (0.2±0.2)^abc^ (0.001±0.01)^c^	(0.9±0.4)^bc^ (0.4±0.2)^cd^ (0.7±0.5)^bcd^	(3.9±0.5)^c^ (5.5±0.4)^a^ (4.6±0.4)^b^	(8.27±0.09)^ab^ (8.2±0.3)^abc^ (8.1±0.3)^abc^
ANOVA	ns	0.0014	≤0.0001	≤0.0001	0.0223
LSD	ns	0.4738	0.5562	0.673	0.3722
NS HN	nsns	ns 0.0006	0.0002 ≤0.0001	0.0053 ≤0.0001	0.0352 NS
NS×HN	ns	NS	0.0001	0.0001	0.0155

Results are expressed as mean value±standard deviation. Different letters indicate significant differences between mean values. NS1=first nutrient solution treatment (depletion), NS2=second nutrient solution treatment (supplementation), NS3=third nutrient solution treatment (correction); significance of factors and their interactions: NS=nutrient solution, HN=number of harvest cycles, NS×HN=nutrient solution×number of harvest cycles; nd=not determined; ns=not significant

Some authors identified a wide spectrum of individual phenols in nettle with high amounts of chlorogenic, 2-O-caffeoylmalic, protocatechuic and α-resorcylic acid as well as kaempferol and rutin (*8*, *9*, *11*, *21*). Repajić *et al.* (*8*) identified a total of 41 compounds, with cinnamic acids as the most abundant polyphenol group in wild nettle leaves, showing that nettle is a very rich source of specific individual phenolic compounds. It is important to point out that plant origin and environmental factors can strongly influence the composition and quantity of individual phenolic compounds in nettle leaves (*8*, *21*).

Hydroxycinnamic acids (such as ferulic, caffeic, sinapic and *p*-coumaric acids), ellagic acid and naringin are a group of compounds known to have potent antioxidant, anti-inflammatory, antimutagenic, anticarcinogenic, antimicrobial and cardioprotective activities (*51*, *52*). Thus, the enhancement of food with phenolic compounds, which are one of the most abundant antioxidants in the human diet, is desirable due to their beneficial medicinal properties for human health. Additional studies to further improve phenolic content of stinging nettle are recommended.

### Photosynthetic pigment content of fresh stinging nettle leaves

In this study, the pigments chlorophyll a (Chl_a), chlorophyll b (Chl_b), total chlorophylls (TCh) and total carotenoids (TCa) were quantified in the stinging nettle leaves (Table 6). Apart from their use as food and pharmaceutical colourants (E140) (*7*), studies have shown that the consumption of chlorophylls and their derivatives may have health-promoting effects and provide protection against a variety of diseases. Carotenoids also have antioxidant effect and are major precursors of vitamin A in humans (*53*). Green leaves are the main plant tissue that is the best source of chlorophyll. Based on the obtained results, it can be observed that the highest TCh and TCa mass fractions were measured in the third harvest regardless of the NS treatment (Table 6), which can be explained by the fact that the plant tries to overcome the stress caused by multiple harvest cycles through the accumulation of antioxidants. The experimental data also showed that the highest mass fraction of all photosynthetic pigments was after the third harvest in NS2 (supplementation). The highest mass fraction of Chl_a (1.21 mg/g) was about 98 % higher than the value observed in NS1 (depletion) in the second (0.62 mg/g) and third harvest cycles (0.61 mg/g). The mass fraction of TCh in treatment NS2 was twofold higher than in NS1 after the third harvest cycle. After each harvest, the plant had to regrow completely, and the increased accumulation of pigments most likely enabled easier photosynthesis and faster growth. The addition of water in NS1 reduced the amount of N, and the N deficiency disturbed the nutrient balance and homeostasis of the plants, which decreased photosynthetic pigment content (*54*). Thus, due to the increased amount of N in NS2 and NS3, which is necessary for photosynthesis and the synthesis of pigments, the accumulation of TCh and TCa probably increased. The studies by Paulauskienė *et al.* (*31*) and Huang *et al.* (*54*) confirm that the content of pigment compounds can strongly depend on abiotic factors such as harvest time and the concentration of nutrient solution, which is in accordance with the present results.

**Table 6:** The mass fraction of photosynthetic pigments of fresh stinging nettle leaves cultivated under different nutrient solution treatments and determined after three harvest cycles

Treatment	*w*/(mg/g)			
Chl_a	Chl_b	TCh	TCa
First harvest				
NS1	(0.80±0.01)^e^	(0.40±0.01)^c^	(1.20±0.02)^e^	(0.24±0.01)^d^
NS2	(0.87±0.02)^c^	(0.36±0.01)^e^	(1.22±0.01)^de^	(0.27±0.01)^c^
NS3	(0.88±0.01)^c^	(0.36±0.01)^e^	(1.23±0.01)^cd^	(0.30±0.01)^b^
Second harvest				
NS1	(0.62±0.01)^g^	(0.38±0.01)^d^	(1.00±0.01)^g^	(0.21±0.01)^f^
NS2	(0.95±0.01)^b^	(0.48±0.01)^b^	(1.43±0.01)^b^	(0.27±0.01)^c^
NS3	(0.84±0.01)^d^	(0.40±0.01)^c^	(1.24±0.01)^c^	(0.27±0.01)^c^
Third harvest				
NS1	(0.61±0.01)^g^	(0.26±0.01)^g^	(0.88±0.01)^h^	(0.23±0.01)^e^
NS2	(1.21±0.01)^a^	(0.62±0.01)^a^	(1.84±0.02)^a^	(0.36±0.01)^a^
NS3	(0.72±0.01)^f^	(0.33±0.01)^f^	(1.05±0.01)^f^	(0.24±0.01)^d^
ANOVA	≤0.0001	≤0.0001	≤0.0001	≤0.0001
LSD	0.0141	0.0112	0.0192	0.0033
NS	≤0.0001	≤0.0001	≤0.0001	≤0.0001
HN	≤0.0001	≤0.0001	≤0.0001	≤0.0001
NS×HN	≤0.0001	≤0.0001	≤0.0001	≤0.0001

Results are expressed as mean±standard deviation. Different letters indicate significant differences between mean values. Chl_a=chlorophyll a, Chl_b=chlorophyll b, TCh=total chlorophylls, TCa=total carotenoids, NS1=first nutrient solution treatment (depletion), NS2=second nutrient solution treatment (supplementation), NS3=third nutrient solution treatment (correction); significance of factors and their interactions: NS=nutrient solution, HN=number of harvest cycles, NS×HN=nutrient solution×number of harvest cycles

### Antioxidant capacity of fresh stinging nettle leaves

Dietary antioxidants prevent oxidative damage caused by increased accumulation of reactive oxygen species (ROS) and nitrogen species (RNS), thus contributing to disease prevention and many other beneficial effects on human health (*55*). Bioactive compounds in leafy vegetables and other foods are responsible for their antioxidant properties. All bioactive substances analysed in this study are well-known important antioxidants. Antioxidant compounds found in plants and foods are hydrophilic to lipophilic, *i.e*. polar and nonpolar substances. Therefore, two different methods were used in this study to determine their antioxidant capacity. The ABTS tests can easily detect both hydrophilic and lipophilic antioxidants, while the FRAP assay is more suitable for hydrophilic antioxidants.

**Fig. 1:**
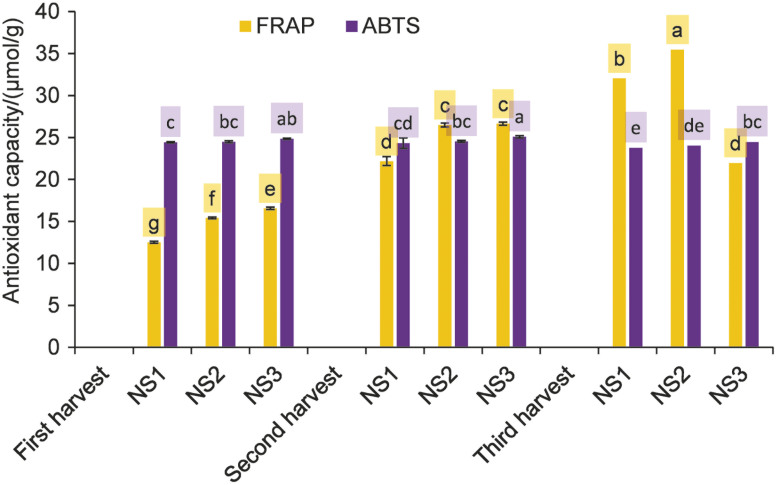
Antioxidant capacity expressed as Trolox equivalents of fresh stinging nettle leaves cultivated under different nutrient solution treatments and subjected to three harvest cycles. Results are expressed as mean±standard deviation. Different letters indicate significant differences between mean values. NS1=first nutrient solution treatment (depletion), NS2=second nutrient solution treatment (supplementation), NS3=third nutrient solution treatment (correction)

The highest value for antioxidant capacity, expressed as Trolox equivalents (TE), measured after NS2 treatment (supplementation) and the third harvest using the FRAP method was 35.47 µmol/g (Fig. 1). This value was 183 % higher than the lowest value (first harvest, NS1). The highest values of AA, total phenolics and photosynthetic pigments were detected after the same harvest cycle and NS treatment when the highest values were also recorded using the FRAP method, confirming these compounds as very powerful antioxidants. Completely different results were observed when using the ABTS assay, where the highest antioxidant capacity was determined in NS3 (correction), after the second harvest, with a value of 25.09 µmol/g. Different treatments with NS, the number of harvest cycles and their combination had a very significant effect on the antioxidant capacity of nettle leaves according to the FRAP method. The ABTS test was affected by the individual factors, but not by their interaction.

Both FRAP and ABTS values were much higher than those in the study by Radman *et al.* (*15*). Stinging nettle also showed the highest antioxidant activity in the ABTS and FRAP assays among the other *Urtica* species (*Urtica urens* and *Urtica membranacea*) in the study by Carvalho *et al.* (*56*). Considering the high antioxidant capacity, which can be further improved by nutritional manipulation with multiple harvest cycles as shown in our study, stinging nettle can be considered as a rich source of bioactive compounds with high antioxidant potential.

## CONCLUSIONS

Considering the challenges and harmful effects that food production leaves on the planet, it is necessary to use sustainable and controlled production techniques. There are many factors throughout the production process that can affect the nutritional composition and quality of food. Proper plant nutrition may be one of the most important first steps in manipulating the nutritional value of the final product resulting in foods with significant biological and pharmacological properties. This study showed that proper nutrition management can improve the nutritional quality of stinging nettle leaves. To achieve the highest contents of ascorbic acid, total phenolics, total flavonoids and photosynthetic pigments, and highest antioxidant capacity, three harvest cycles and supplementation with nutrient solution are recommended. Based on the results, a chemical analysis of the nutrient solution after each harvest is not necessary; it is enough to add the same initial solution to ensure the highest content of bioactive compounds. However, to ensure the highest content of essential macronutrients important for proper body function (Ca and Mg), depletion of nutrient solution by adding water before the second harvest proved to be the best treatment. Also, for the consumption of nettle as a green leafy vegetable, it is recommended to cultivate it under controlled conditions to ensure the safety and quality of the plant material available throughout the whole year.
